# Diversity and cross-species transmission of viruses in a remote island ecosystem: implications for wildlife conservation

**DOI:** 10.1093/ve/veae113

**Published:** 2024-12-14

**Authors:** Rebecca K French, Sandra Anderson, Kristal Cain, Andrew Digby, Terry C Greene, Colin M Miskelly, Chris G Muller, Michael W Taylor, Kākāpō Recovery Team, Jemma L Geoghegan, Edward C Holmes

**Affiliations:** Department of Microbiology and Immunology, University of Otago, Dunedin 9016, New Zealand; School of Medical Sciences, The University of Sydney, Sydney, NSW 2006, Australia; School of Biological Sciences, University of Auckland, Auckland 1010, New Zealand; School of Biological Sciences, University of Auckland, Auckland 1010, New Zealand; Department of Conservation, Kākāpō Recovery Team, Invercargill 9810, New Zealand; Department of Conservation, Christchurch 8011, New Zealand; Museum of New Zealand Te Papa Tongarewa, Wellington 6011, New Zealand; Wildbase, School of Veterinary Sciences, Massey University, Palmerston North 4410, New Zealand; Zoology and Ecology Group, Massey University, Palmerston North 4442, New Zealand; School of Biological Sciences, University of Auckland, Auckland 1010, New Zealand; Kākāpō Recovery Team‡, Department of Conservation, Invercargill 9810, New Zealand; Department of Microbiology and Immunology, University of Otago, Dunedin 9016, New Zealand; Institute of Environmental Science and Research, Wellington 5022, New Zealand; School of Medical Sciences, The University of Sydney, Sydney, NSW 2006, Australia

**Keywords:** birds, virus, evolution, metatranscriptomics, cross-species transmission

## Abstract

The ability of viruses to emerge in new species is influenced by aspects of host biology and ecology, with some taxa harbouring a high diversity and abundance of viruses. However, how these factors shape virus diversity at the ecosystem scale is often unclear. To better understand the pattern and determinants of viral diversity within an ecosystem, and to describe the novel avian viruses infecting an individual avian community, we performed a metagenomic snapshot of the virome from the entire avian community on remote Pukenui/Anchor Island in Aotearoa New Zealand. Through total RNA sequencing of 18 bird species, we identified 50 avian viruses from 9 viral families, of which 96% were novel. Of note, passerines (perching birds) exhibited high viral abundance and diversity, with viruses found across all nine viral families identified. We also identified numerous viruses infecting seabirds on the Island, including megriviruses, hepaciviruses, and hepatoviruses, while parrots exhibited an extremely low diversity of avian viruses. Within passerines, closely related astroviruses and hepatoviruses, and multiple identical hepe-like viruses, were shared among host species. Phylogenetic reconciliation analysis of these viral groups revealed a mixture of co-divergence and cross-species transmission, with virus host-jumping relatively frequent among passerines. In contrast, there was no evidence for recent cross-species virus transmission in parrots or seabirds. The novel pegiviruses and a flavivirus identified here also pose intriguing questions regarding their origins, pathogenicity, and potential impact on vertebrate hosts. Overall, these results highlight the importance of understudied remote island ecosystems as refugia for novel viruses, as well as the intricate interplay between host ecology and behaviour in shaping viral communities.

## Introduction

Several barriers must be overcome for a virus to successfully transmit and persist in a new host species. The virus must first have the opportunity to encounter the new host, whether through direct inter-species interactions, via vectors, or through indirect transmission via the environment. The virus must then successfully infect the host by replicating and evading the host immune response for a sufficient length of time to enable onward transmission (Reperant et al. 2012). Emergence success is strongly associated with the degree of phylogenetic relatedness between the original and the new host—because cell systems including cell receptors evolve as their hosts do—and by how specialized the virus is to the original host (Haller et al. 2014). Finally, the virus must establish sustained transmission in the new host population, which may be constrained by ecological factors such as a low population density that limits transmission opportunities (French and Holmes 2020). Aspects of host biology and ecology are therefore central to the successful cross-species transmission of viruses (French et al. 2023), such that some species will necessarily harbour a greater diversity and abundance of viruses than others (Luis et al. 2015). For example, some bat species carry a high diversity of viruses, which has been attributed to such factors as multispecies roosting that enables frequent interspecific transmission (Ruiz-Aravena et al. 2022), and host immune responses that allow tolerance to viral infection (Letko et al. 2020).

In birds, both host phylogeny and ecology play pivotal roles in shaping the species range of avian influenza virus (Huang et al. 2019, Wille et al. 2023, Yin et al. 2024). For example, in waterfowl and shorebirds, both geographic location and influenza A virus infection status are important determinants of viral abundance and diversity (Wille et al. 2018). However, beyond avian influenza virus, differences in viral abundance and diversity between bird groups remain largely unexplored, although it is likely that closely related bird taxa and those that engage in frequent interspecific interactions will share more viruses than those that occupy differing ecological niches and rarely interact (French et al. 2023).

Accurately comparing the viral diversity between different hosts taxa can be challenging. Differences in sampling method, sequencing method and depth, as well as bioinformatics pipelines (such as the diversity cut-offs to assign novel viruses), will impact the number and types of viruses discovered (Tangherlini et al. 2016, Cantalupo and Pipas 2019, Cobbin et al. 2021). Additionally, the viral community within host species will vary in time and space, particularly among seasons and across different ecological communities (Gaidet et al. 2012, Wille et al. 2018). As such, the most meaningful comparisons of viral diversity across different host species necessitate that they be sampled using the same methods, in the same geographic area, and within a given timeframe.

New Zealand’s geographic isolation has resulted in the divergence and radiation of many distinct avian lineages, as well as a high number of endemic bird species (Wallis and Trewick 2009). It is plausible that this has also led to the evolution of novel viruses, which may in turn have implications for our understanding of the factors that shape virus ecology and emergence. Until recently, much of our knowledge of viruses in New Zealand birds was derived from studies of pathogenic viruses, with little known about the viruses naturally present in avian communities in the absence of overt disease. Fortunately, a number of studies have now begun to address this knowledge gap, revealing a diverse assemblage of novel viruses in New Zealand passerines (Custer et al. 2022, French et al. 2022a, 2022b, Grimwood et al. 2024). Despite this, little is still known about the viromes of New Zealand birds other than passerines, and whether other bird groups host a similarly diverse viral assemblage.

Herein, we provide a snapshot of the virome from the entire avian community on Pukenui/Anchor Island—a small island nature reserve (11.4 km^2^) that is part of Fiordland National Park, New Zealand. We collected cloacal swabs from individuals of each bird species present on the Island over a 1-month period, ensuring that comparisons between species were effectively made in ‘real time’. Using total RNA sequencing (i.e. metatranscriptomics), we compared the viral diversity of different host species and described the novel avian viruses infecting these hosts.

## Methods

This research was conducted under the Department of Conservation Wildlife Act Authority authorization number 86173-FAU and authority for research and/or collection of material on public conservation land authorization number 86172-RES, and had ethics approval from the University of Auckland (reference number 002198).

### Study location

Pukenui/Anchor Island in Dusky Sound, Fiordland, New Zealand (Supplementary Fig. S1) lies within the expansive and largely uninhabited Fiordland National Park, which spans more than 12 000 km^2^ on the southwest coast of the South Island. Situated >80 km from the nearest township by air, this Island is an important habitat for endangered native species, including the Kākāpō (*Strigops habroptila*) and Te Kakahu/Chalky Island skink (*Oligosoma tekakahu*, the only reptile on the Island) after the successful eradication of invasive mammals in the early 2000s. It also serves as a nesting site for forest-dwelling seabirds, such as the Tawaki/Fiordland crested penguin (*Eudyptes pachyrhynchus*). There are no amphibians or terrestrial mammals inhabiting the Island. The Island’s temperate rainforest is dominated by beech and podocarp (conifer) trees, with an understory rich in shrubs, vines, and mosses. Currently, the only known non-native species permanently residing on the Island is the invasive German wasp (*Vespula germanica*).

### Sample collection and RNA extraction

The methods used for sample collection, RNA extraction and sequencing, quality control, assembly, and virus identification are described in detail in French et al. (2023). Briefly, between February and March 2021, 119 individual birds from 18 species were caught on Pukenui/Anchor Island using mist-netting and hand-capture depending on the species in question (Table 1). A cloacal swab was taken from each individual bird. Total RNA was extracted from the swabs using the RNeasy plus mini extraction kit (Qiagen) and QIAshredders (Qiagen). Complementary DNA libraries were prepared using the Stranded Total RNA Prep with Ribo-Zero Plus (Illumina), then sequenced using Illumina NovaSeq via Auckland Genomics Ltd, University of Auckland, with one library per bird species. A single blank negative control library (i.e. a sterile water and reagent mix) was also sequenced.

**Table 1.** Details of each RNA-sequencing library

Common name	Latin name	Order	Family	Number per library	Total reads (million)	No. of avian virus reads	Avian virus (%)	Avian virus RPM
Little spotted kiwi/kiwi pukupuku	*Apteryx owenii*	Apterygiformes	*Apterygidae*	3	197	0	0	0
New Zealand falcon/kārearea	*Falco novaeseelandiae*	Falconiformes	*Falconidae*	1	202	0	0	0
Grey warbler/riroriro	*Gerygone igata*	Passeriformes	*Acanthizidae*	9	227	15 289 184	6.7	67 459
Tieke/South Island saddleback	*Philesturnus carunculatus*	Passeriformes	*Callaeidae*	10	205	1122	0.0005	5
Fernbird/mātātā	*Poodytes punctatus*	Passeriformes	*Locustellidae*	4	190	221 646	0.1	1168
Bellbird/korimako	*Anthornis melanura*	Passeriformes	*Meliphagidae*	10	229	1316	0.0005	6
Brown creeper/pīpipi	*Mohoua novaeseelandiae*	Passeriformes	*Mohouidae*	4	210	134 022	0.06	639
Mohua/yellowhead	*Mohoua ochrocephala*	Passeriformes	*Mohouidae*	10	188	21 856	0.01	116
South Island robin/kakaruai	*Petroica australis*	Passeriformes	*Petroicidae*	10	222	281 512	0.1	1270
Tomtit/miromiro	*Petroica macrocephala*	Passeriformes	*Petroicidae*	5	214	655 410	0.3	3069
Fantail/pīwakawaka	*Rhipidura fuliginosa*	Passeriformes	*Rhipiduridae*	10	250	297 670	0.1	1191
Tītī/sooty shearwater	*Ardenna grisea*	Procellariiformes	*Procellariidae*	10	196	4034	0.02	21
Kōrure/mottled petrel	*Pterodroma inexpectata*	Procellariiformes	*Procellariidae*	10	243	1,896	0.0008	8
Kākāriki/yellow-crowned parakeet	*Cyanoramphus auriceps*	Psittaciformes	*Psittaculidae*	6	240	0	0	0
Kākā	*Nestor meridionalis*	Psittaciformes	*Strigopidae*	1	215	438	0.0002	2
Kākāpō	*Strigops habroptila*	Psittaciformes	*Strigopidae*	10	242	0	0	0
Tawaki/Fiordland crested penguin	*Eudyptes pachyrhynchus*	Sphenisciformes	*Spheniscidae*	5	226	572	0.0002	3
Morepork/ruru	*Ninox novaeseelandiae*	Strigiformes	*Strigidae*	1	0.6	0	0	0

RPM = avian (vertebrate) virus reads per million from rRNA-depleted libraries to the nearest whole number. Number per library = number of individual birds sampled. Total reads is rounded to the nearest million.

### Contig assembly and virus identification

Following quality control, sequence reads were *de novo* assembled into contiguous sequences (contigs), which were identified by comparing the assembled contigs to the National Center for Biotechnology Information (NCBI) nucleotide database (nt) and nonredundant protein database (nr) using BLAST (Basic Local Alignment Search Tool)-based methods. Contigs were retained that had hits to viruses and an open reading frame (ORF) greater than 300 nt for nr hits. To prevent false-positive virus identifications, sequence similarity cut-off values of 1 × 10^−5^ and 1 × 10^−10^ were used for the nt and nr databases, respectively. Virus abundance was estimated by mapping the reads back to the contigs. Viral sequences with total read counts that were<0.1% of the read count in another library and >99% identical at the nucleotide level were assumed to be contamination due to index-hopping from another library and were removed. Similarly, any virus found in the blank negative control library was assumed to represent reagent contamination and was removed from all libraries and analyses.

The likely host of origin of all viruses detected was inferred based on their phylogenetic similarity to other viruses. Specifically, we assumed that novel viruses that were closely related to known vertebrate viruses were similarly vertebrate-associated and, hence, likely avian viruses. These likely avian viruses were subjected to additional evolutionary analyses. Other viruses (i.e. those phylogenetically distinct from known vertebrate viruses) were assumed to be more likely associated with host diet, microbiome, or environment.

### Alpha diversity analysis

Alpha diversity was analysed using richness (i.e. the number of avian viral species), abundance (expressed as sequence reads per million), and the Shannon diversity index (a measure of community species diversity; Shannon 1948). To avoid comparisons between taxonomic groupings with a single member (i.e. one host species), two species (little spotted kiwi/kiwi pukupuku *Apteryx owenii* and New Zealand falcon/karearea *Falco novaeseelandiae*) that are in distinct phylogenetic orders were excluded from the alpha diversity analysis. To improve the evenness of sample sizes across the groups, the two procellariiform species (Tītī/sooty shearwater *Ardenna grisea* and Kōrure/mottled petrel *Pterodroma inexpectata*) were grouped with the one penguin species (Tawaki/Fiordland crested penguin) to create the “seabird” group as they are sister clades (Cole et al. 2022). Avian host species were then classified into three groups according to host taxonomy—passerines, seabirds, and parrots. Linear models and ANOVAs were used to test for differences in abundance and Shannon diversity across the three groups, and generalized linear models (GLM) were used to test for differences in richness (count data, Poisson distribution). Information on the structure of each model is provided in Supplementary Table S1. We verified that none of the GLMs were overdispersed using the ‘dispersiontest’ function in the R package AER (v 1.2-14) (Kleiber et al. 2020). We also tested whether differences in abundance and Shannon diversity were affected by the number of individual hosts in each library and the evolutionary distinctiveness of each host species using Evolutionary Distinctiveness (ED) scores with the method by McClure et al. (2023). ED scores measure the isolation of a species in its phylogenetic tree or the relative contribution of a species to phylogenetic diversity (McClure et al. 2023). Hence, a species with a higher ED score is more phylogenetically isolated and evolutionarily distinct than a species with a lower ED score. Bar graphs were created using ggplot2 (3.3.5) (Wickham 2011).

### Phylogenetic analysis

Virus amino acid sequences were aligned with representative sequences from the same viral genus or family obtained from NCBI/GenBank. Representative sequences were selected to include a range of diversity within each genus or family by sampling a small number of sequences from each major subgroup or clade, and including all sequences closely related to the viruses found in our study. Sequences were aligned using MAFFT (7.402) (Katoh and Standley 2013) employing either the E-INS-i or L-INS-i algorithms (Supplementary Table S2), and trimmed using TrimAl (1.4.1) (Capella-Gutiérrez et al. 2009). The maximum likelihood method in IQ-TREE (1.6.12) was used to estimate individual phylogenetic trees for each viral family or genus, with the best-fit substitution model determined by the program and node robustness assessed by employing the approximate likelihood ratio test with 1000 replicates (Nguyen et al. 2015). Where similar sequences were found in the same library, they were assumed to represent different viral species (rather than intra-specific viral diversity) if they met either of the following criteria: (i) the aligned sequences were overlapping and <90% identical at the amino acid level, or (ii) the aligned sequences were non-overlapping but were separated by significant bootstrap values in the phylogenetic tree. Phylogenetic trees were visualized using APE (5.4) (Paradis et al. 2019) and ggtree (2.4.1) (Yu et al. 2017) in R (4.0.5) (R Core Team 2021).

### Phylogenetic reconciliation analysis

To determine the dual contributions of cross-species virus transmission (i.e. host-switching) and co-divergence to the evolutionary history of passerine viruses, we conducted a phylogenetic reconciliation analysis on the three viral groups that had viruses from more than two host species sampled in this study: the genera *Avastrovirus, Hepatovirus*, and hepe-like viruses. Accordingly, we mapped the phylogenetic trees of avian viruses against those of their avian hosts to calculate the relative proportions of four different co-evolutionary events: co-divergence, duplication, extinction, and host-switching. Co-divergence is observed when the topology of virus phylogeny matches that of the host phylogeny; duplication refers to a virus lineage evolving independently from the host lineage; extinction refers to the death of a virus lineage; and host-switching denotes horizontal transmission of viruses between different species. Host cladograms were created from the Open Tree of Life using the R package rotl (Michonneau et al. 2016). The relative frequencies of cross-species virus transmission versus virus–host co-divergence were quantified using eMPRess (Santichaivekin et al. 2021), which employs a maximum parsimony approach to align the virus phylogeny with the host phylogeny. The event costs were set according to the established literature: duplication, host-jumping, and extinction events were assigned a cost of 1.0, while virus–host co-divergence was assigned a cost of zero, as it can be considered the null event (Geoghegan et al. 2017, Shi et al. 2018).

## Results

We sequenced the Pukenui/Anchor Island avian community at depth. Overall, total RNA sequencing of 18 libraries (one per bird species) yielded 3.8 billion reads, ranging extensively from 600 000 to ∼250 million reads per library. One library (morepork/ruru *Ninox novaeseelandiae*) had considerably fewer reads than the other libraries (Table 1) and was therefore removed from all analyses. A range of 0–6.7% of the rRNA-depleted reads in the libraries were identified as associated with vertebrate viruses: a mean of 0.04% vertebrate viruses per library, excluding the very high number of viruses in the grey warbler (riroriro) library (6.7%) that was associated with a highly abundant astrovirus (see further).

**Figure 1.**
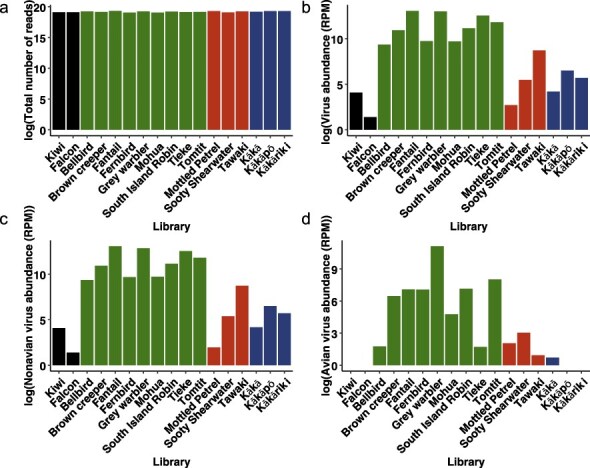
(a) Total number of reads in each library. (b) Abundance of viral reads. (c) Abundance of non-avian viral reads. (d) Abundance of avian viral reads. For all graphs the *y*-axis is a log scale. For panels (b–d), the abundance is expressed as RPM. Green = passerine, red = seabird, blue = parrot, black = other.

Based on phylogenetic analysis and previous studies, we identified 50 viruses from 9 viral families that we considered highly likely to have infected the particular avian host sampled (Supplementary Table S3). Although the total number of reads between libraries was very consistent (Fig. 1a, excluding the morepork library), the number of viruses detected varied markedly between host species (Fig. 1b-d). While all libraries had viruses, four bird species—little spotted kiwi, New Zealand falcon, Kākāpō, and Kākāriki—had no known avian-infecting viruses. In contrast, all the passerine bird species had at least one avian host-associated virus, with the fantail and grey warbler having the most viruses identified in the study (11 from 6 viral families and 13 from 4 viral families, respectively).

The abundance of avian viruses similarly varied considerably, from fernbird chaphamaparvovirus at 0.03 reads per million (RPM) to avian astrovirus 6 in the grey warbler at >37 000 RPM (Supplementary Table S3), although as our study utilized total RNA sequencing there may be systematic differences in abundance between RNA and DNA viruses. Passerines generally had a relatively high abundance of viral reads, particularly the grey warbler: >67 000 RPM in total from 13 virus species, including multiple astroviruses as well as a high abundance atadenovirus (7877 RPM) and hepe-like virus (4651 RPM). Overall, viruses belonging to the families *Astroviridae* and *Hepeviridae* generally had high abundances, with an average of 4600 (± 3053 SE) and 732 (± 316 SE) RPM, respectively (Supplementary Table S3).

**Figure 2.**
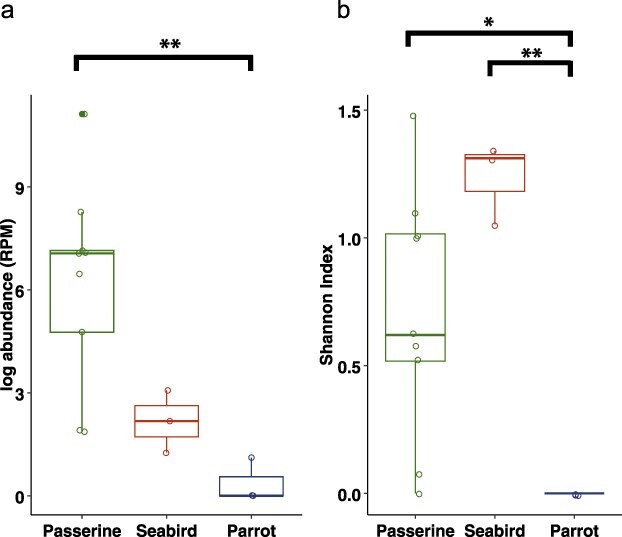
Boxplots showing the differences in (a) log viral abundance (RPM) and (b) Shannon index across the three species groups. Brackets show *P*-value significance, * <.05, ** <.01. Raw data (jittered) is shown using empty circles.

Of note, passerines had a significantly higher abundance of avian host-associated viruses than parrots (*P* = .006, Fig. 2a; Supplementary Table S3). In addition, both passerines and seabirds had significantly higher avian host-associated virus richness (number of species, *P* = .007 estimate = 2.73 ± SE 1 and *P* = .01 estimate = 2.57 ± SE 1.04, respectively; Supplementary Table S3) and Shannon index than parrots (*P* = .02 and *P* = .003, respectively, Fig. 2b; Supplementary Table 3). Avian-associated virus richness declined significantly (*P* = .01, estimate = -0.06 ± SE 0.02; Supplementary Table S3) according to the ED of the hosts (ED score). However, this was heavily influenced by the Kākā and Kākāpō, which had very high ED scores and very low viral richness. When these taxa were removed, the ED score was a strongly nonsignificant factor (*P* = .7, estimate = −0.02 ± SE 0.05). The ED score was not a significant factor for viral abundance and Shannon index (*P* = .15, adjusted *R*^2^ = 0.08 and *P* = .1, adjusted *R*^2^ = 0.14, respectively; Supplementary Table S3). Similarly, the number of individuals sampled per host species was not a significant factor across all comparisons (*P* > .05; Supplementary Table S3). As the grey warbler had a much greater abundance of avian viruses than all other libraries, we repeated all models with this library removed; this did not change the results and the passerines still had greater viral abundance and Shannon index than parrots (*P* = .003, adjusted *R*^2^ = 0.53 and *P* = .02, adjusted *R*^2^ = 0.58, respectively).

Non-vertebrate viruses (i.e. those likely associated with host diet, microbiome or environment, excluding bacteriophage) were frequently shared between hosts, with over 900 viruses shared (i.e. with >90% amino acid identity) between host libraries. The vast majority of these viruses (96%) were shared among different passerine species, and comprised viruses from the ‘unclassified *Picornavirales*’ (291 viruses), *Iflaviridae* (128 viruses), *Solemoviridae* (93), *Dicistroviridae* (85), and *Tombusviridae* (75). Indeed, only seven viruses were shared among seabird species (from the *Phasmaviridae* and *Totiviridae* families) and there were no viruses shared between parrot species (Supplementary Table S4). There were also a few instances of the same non-vertebrate virus shared among distantly related species groups, including between passerines and parrots (five virus species from the *Mayoviridae* and *Narnaviridae* families), passerines and seabirds (15 virus species from the *Picornaviridae, Tombusviridae, Totiviridae*, and unclassified *Picornavirales*) and seabirds and parrots (three virus species from the *Narnaviridae* and *Totiviridae*) (Supplementary Table S4). In contrast, most avian virus species were unique to the host in question. We now describe the different groups of avian viruses in turn.

### Single-stranded DNA viruses

We identified one very short sequence (length 323 nucleotides, 107 amino acids) of a virus belonging to the *Anelloviridae* in the fantail (Supplementary Fig. S2a). This fragment, representing part of the VP1 gene, was most closely related (67% amino acid identity) to Gyrovirus 11 found in a South American passerine—the ferruginous-backed antbird (*Myrmoderus ferrugineus*) (Truchado et al. 2019). In addition, fragments of the nonstructural proteins from two novel parvoviruses of the genus *Chaphamaparvovirus* were found in the Kākā and fernbird (Supplementary Table S3, Supplementary Fig. S2b). The Kākā library contained a virus most closely related (although with only 55% amino acid identity) to duck parvovirus (*Dependoparvovirus anseriform 1*) found in commercially farmed ducks in Australia, while the virus in the fernbird fell within a clade of presumed dietary viruses from predatory African army ants (*Dorylus* spp.) (Fritz et al. 2023).

### Double-stranded DNA viruses

The complete genome of a novel adenovirus was identified in the grey warbler, and present at very high abundance (7877 RPM, Supplementary Fig. S2c). Phylogenetic analysis of the DNA polymerase gene showed this virus belonged to the genus *Atadenovirus*, that infects a wide variety of vertebrates, and was most closely related (64% amino acid identity) to Passerine adenovirus 1 (Athukorala et al. 2020), found in faeces from the Eastern spinebill (*Acanthorhynchus tenuirostris*).

### Positive-sense single-stranded RNA viruses

**Figure 3.**
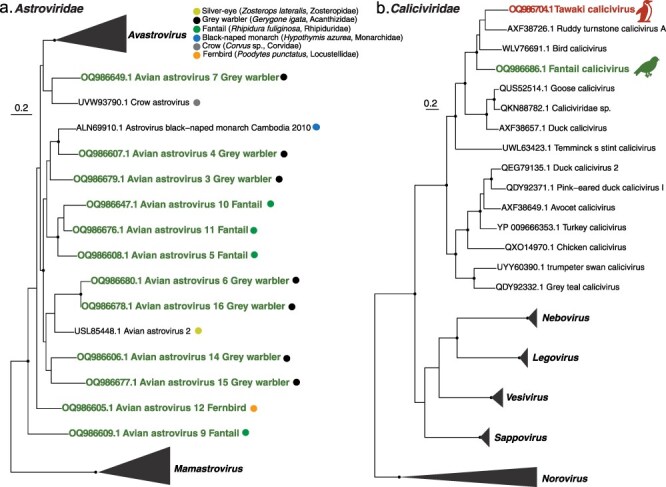
(a) Phylogeny of the *Astroviridae* (representative viruses only, *n* = 33 including collapsed clades) based on the nonstructural polyprotein (alignment length of 374 amino acids post-trimming). Coloured circles depict the host species for each virus found as shown in the key (for passerine astrovirus 1–4 the host species is unknown). (b) Phylogeny of the *Caliciviridae* (representative viruses only, *n* = 25 including collapsed clades) using the polyprotein gene (alignment length of 1693 amino acids post-trimming). Coloured text denotes viruses found in this study, with related viruses shown in black. Black circles on nodes show bootstrap support values of >90%. Branches are scaled according to the number of amino acid substitutions per site, shown in the scale bar. The trees are midpoint rooted for purposes of clarity only. Silhouettes were adapted from Microsoft 365 icons.

We detected 12 astroviruses in 6 different passerine libraries (Fig. 3a; Supplementary Table S3). These viruses all fell within a clade of astroviruses found in passerine species and were related to passerine astroviruses 1–4 with 48–70% amino acid identity. In turn, this passerine clade was the sister group to viruses of the genus *Avastrovirus* associated with avian hosts, such that the clade of passerine astroviruses identified here should also be placed within the genus *Avastrovirus*. Within the passerine-infecting clade there was minimal clustering according to the host, particularly for the viruses found in the grey warbler (Fig. 3a).

We identified one calicivirus in the fantail that was most closely related (63% amino acid identity) to another calicivirus we found in the Tawaki (Fig. 3b). Interestingly, these viruses fell into a clade of avian-associated caliciviruses found in chickens and waterbirds, and were most closely related (60–67% amino acid identity) to bird calicivirus found in a cormorant in China.

**Figure 4.**
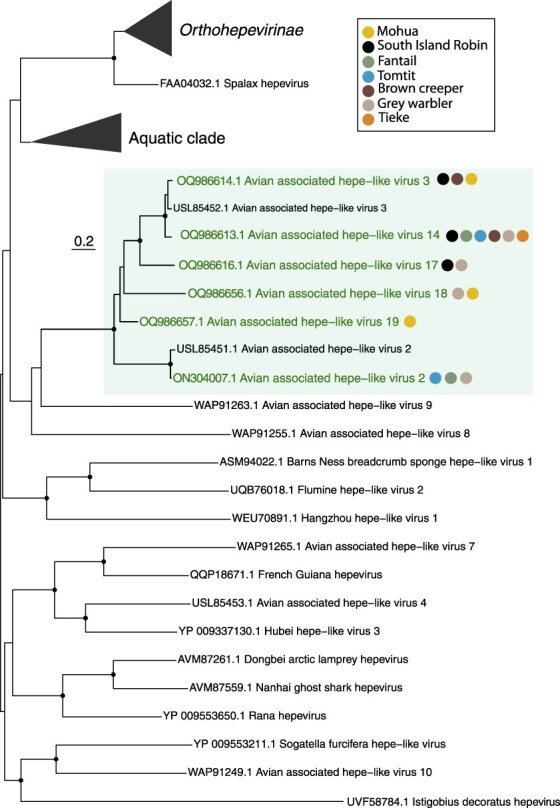
Phylogenetic analysis of the Hepeviridae (representative viruses only, *n* = 67 including collapsed clades) based on the nonstructural polyprotein (alignment length of 762 amino acids post-trimming). Green coloured text denotes viruses found in this study, while related viruses are shown in black. In the avian clade, coloured circles show the host species for each virus found in the study as shown in the key. Black circles on nodes show bootstrap support values of >90%. Branches are scaled according to the number of amino acid substitutions per site, shown in the scale bar. The tree is midpoint rooted for purposes of clarity only. Silhouettes were adapted from Microsoft 365 icons.

We identified four novel hepe-like viruses in passerine libraries that formed a monophyletic group with other avian hepe-like viruses found in New Zealand passerines (French et al. 2022a), with 47–88% amino acid similarity. This avian clade falls basal to the *Orthohepevirinae* subfamily (albeit with low bootstrap confidence) within a wider group of hepe-like viruses predominantly found in birds and fish (Fig. 4). We also identified two viruses previously discovered in passerines in the South Island of New Zealand (French et al. 2022a): *Avian associated hepe-like virus 2* in three different passerine species, that shared 95% amino acid identity with a virus previously found in a bellbird (French et al. 2022a), and *Avian associated hepe-like virus 3* in three different passerine species, that shared 92% amino acid identity with a virus previously found in a dunnock (*Prunella modularis*) (Fig. 4).

**Figure 5.**
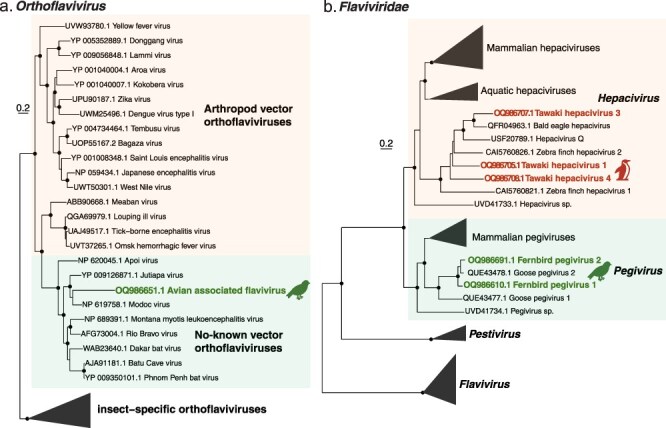
(a) Phylogenetic analysis of the genus *Orthoflavivirus* (*Flaviviridae*) (representative viruses only, *n* = 36 including collapsed clade) based on the polyprotein (alignment length 2486 amino acids post trimming). (b) Phylogenetic analysis of the *Flaviviridae* (representative viruses only, *n* = 139 including collapsed clades) based on the polyprotein (alignment length 1562 amino acids post trimming). Green/red text and icons denote the novel viruses found in this study. Related viruses are shown in black. Black circles on nodes show bootstrap support values of >90%. Branches are scaled according to the number of amino acid substitutions per site, shown in the scale bar. The tree is midpoint rooted for purposes of clarity only. Silhouettes were adapted from Microsoft 365 icons.

Of note, given their common disease association, we identified one short fragment of a flavivirus (141 amino acids in length) in the brown creeper that fell into the clade of so-called ‘no-known vector’ orthoflaviviruses with high bootstrap support but a relatively long branch length (Fig. 5a). This sequence was most closely related to *Modoc virus* (a no-known vector flavivirus associated with rodents), exhibiting 66% amino acid identity, although it was only found at low abundance (1.36 RPM).

We similarly identified two pegiviruses in the fernbird that were relatively closely related (85–93% amino acid identity) to *Goose pegivirus 2* (Fig. 5b). Finally, we identified three very low abundance (0.19 RPM) hepaciviruses in the Tawaki that fell within a clade of avian viruses, including *Bald eagle hepacivirus* and two zebra finch hepaciviruses (Fig. 5b). Although Tawaki hepacivirus 3 was separated from the other two Tawaki hepaciviruses by high bootstrap values (86% and 98%), and was most closely related to *Bald eagle hepacivirus*, the similar abundances and short lengths of the Tawaki sequences may mean that they are in fact variants of the same virus species.

**Figure 6.**
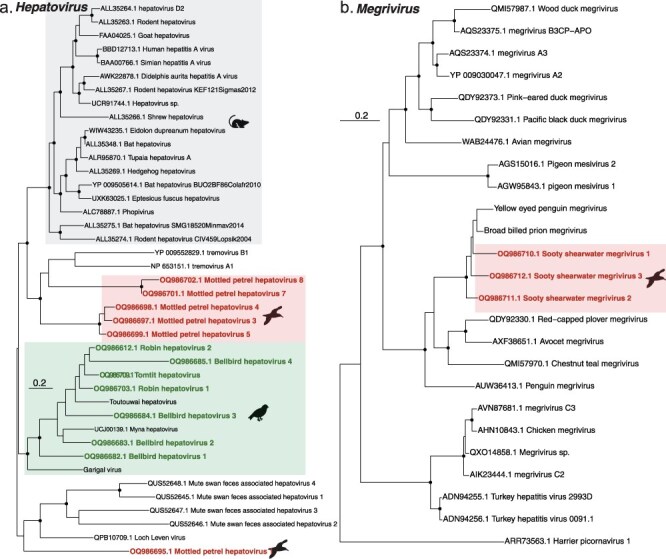
Phylogenetic analysis of two *Picornaviridae* genera: (a) the genus *Hepatovirus* (representative viruses only, *n* = 44) based on the polyprotein (alignment length 1699 amino acids post-trimming). (b) The genus *Megrivirus* (representative viruses only, *n* = 27) based on the polyprotein (alignment length 2063 amino acids). Shading shows clustering according to host; grey = mammalian-infecting viruses, green = passerine-infecting viruses, red = mottled petrel-infecting viruses. Coloured text and black icons denote viruses found in this study, while related viruses are shown in black. Black circles on nodes show bootstrap support values of >90%. Branches are scaled according to the number of amino acid substitutions per site, shown in the scale bar. The tree is midpoint rooted for purposes of clarity only. Silhouettes were adapted from Microsoft 365 icons.

We identified viruses within the *Picornaviridae* that belonged to two different genera. Thirteen viruses fell within the genus *Hepatovirus*: four in the bellbird, six in the mottled petrel, two in the robin, and one in the tomtit. Most of these viruses clustered within the wider avian basal clade (Fig. 6a), with the passerine viruses grouping closely with *Toutouwai hepatovirus* found in the North Island robin (French et al. 2022b), with up to 71% amino acid similarity. The mottled petrel viruses fell into two distinct groupings: one was most closely related to hepatoviruses found in the ruddy turnstone (*Arenaria interpres*) and mute swan (*Cygnus olor*) in the avian basal clade, with 59% amino acid identity, while the other was more closely related to members of the genus *Tremovirus* (albeit with relatively low amino acid identities of 35–58%), and the mammalian hepatoviruses. We also identified three megriviruses in the sooty shearwater that grouped with two other megriviruses found in New Zealand (Fig. 6b): in the broad-billed prion (*Pachyptila vittata*, 85% amino acid identity) and yellow-eyed penguin (*Megadyptes antipodes*, 87% amino acid identity) (Wierenga et al. 2023). The presence of multiple closely related viruses within the same host species is indicative of intraspecific differentiation.

### Double-stranded RNA viruses

Finally, we identified segments from five viruses belonging to the family *Sedoreoviridae* (Supplementary Fig. S2d). This included partial VP1 genes from a rotavirus G virus in the Tawaki library, that were most closely related (71% amino acid identity) to *Ruddy turnstone rotavirus* found in an Australian wading bird (Wille et al. 2018), and were also closely related to two viruses found in ducks. These viruses fell within the wider G clade of rotaviruses found in birds. We also identified partial VP7 genes from two banna-like viruses in the fantail and grey warbler that fell within a clade of vector-borne viruses with banna virus, and partial VP1 genes of two seadorna-like viruses in the fantail, most closely related to *Liao ning virus* (46% amino acid identity), which is also vector-borne.

### Phylogenetic reconciliation

**Figure 7.**
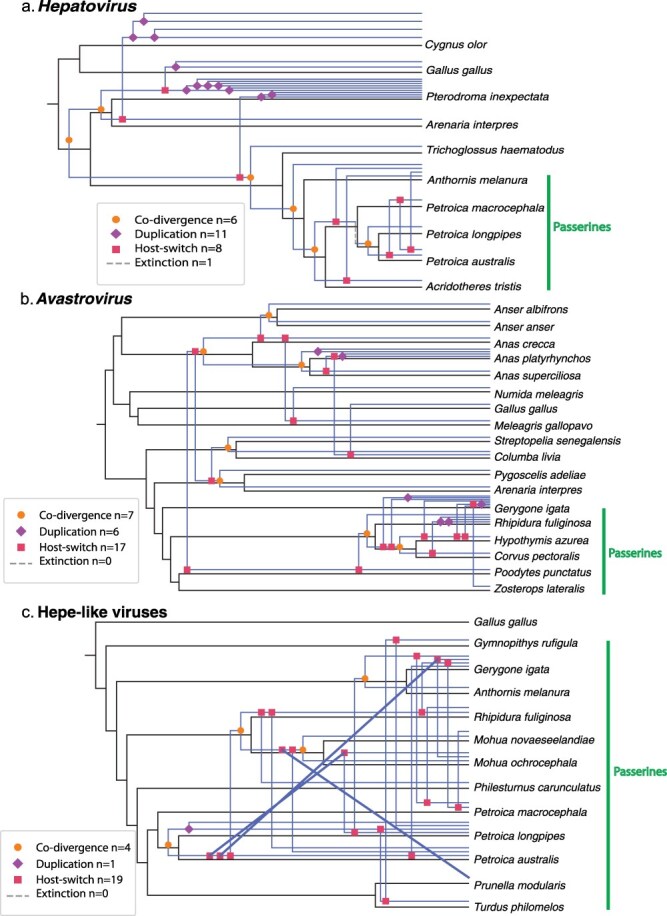
Median maximum parsimony reconciliation phylogenies for avian viruses in the *Hepatovirus* (a), *Avastrovirus* (b) genera, and the hepe-like viruses (c) group. The host phylogenies are shown in black with binomial names of each host in black text. The viral phylogenies are overlaid in blue, with no tip labels. Symbols on the nodes of the viral phylogenies denote the evolutionary event inferred.

A reconciliation analysis of host and virus phylogenies revealed that the *Avastrovirus, Hepatovirus*, and hepe-like viruses all had evolutionary histories that included both co-divergence and cross-species transmission events (Fig. 7). Cross-species transmission accounted for 55% of events, while co-divergence accounted for 21%, duplication 23%, and extinction 1%. Within the passerine virus clades, cross-species transmission accounted for the majority of evolutionary events (68%), while co-divergence accounted for 19% of events, duplication 11%, and extinction 2%. Hence, while there is some evidence for virus–host co-divergence in the evolutionary history of these viruses, cross-species transmission has played a more important role, particularly within the passerine virus clades.

## Discussion

Despite its relative isolation, we found a high diversity of viruses in the avian community on Pukenui/Anchor Island. All but two of the 50 likely vertebrate viral species identified were novel (96%), demonstrating the large diversity of undiscovered viruses present in remote areas such as Fiordland, New Zealand. However, we also identified some viruses that were closely related to those previously found on the North and South Islands of New Zealand (French et al. 2022a, 2022b, Wierenga et al. 2023) and the Chatham Islands (Grimwood et al. 2024), located 800 km east of the South Island and 1350 km east of Pukenui/Anchor Island. Accordingly, these results suggest there has been viral movement between these islands, although without wider sampling, the extent and direction of transmission will be difficult to determine.

Our study also provided some evidence for cross-species virus transmission on Pukenui/Anchor Island. A case in point were the hepe-like viruses from passerine species that contained viral sequences with >90% amino acid identity among different host species. Although it is possible that these hepe-like viruses are not infecting birds themselves given their phylogenetic position among other metagenomically discovered viruses of uncertain origin, they were not detected in any invertebrates and plants on Pukenui/Anchor Island (French et al. 2023), with the exception of a much smaller sequence fragment in a cockroach (*Blattodea* sp.) and German wasp (*Vespula germanica*), both of which are common carrion eaters. Similarly, we identified many other viruses in passerines that clustered together into closely related clades and without the clear phylogenetic patterns that would indicate virus-host co-divergence. For example, although the avastro-like viruses in the *Astroviridae* do cluster at the host order level (with a well-supported clade of closely related viruses found only in passerines), they show no clustering according to host genus or family. In particular, multiple grey warbler astroviruses were found in different phylogenetic positions within the clade, and were often more closely related to astroviruses from other host species. Similarly, there is a well-supported clade of hepatoviruses only found in passerines, but with no clustering at the host genus or family levels, and bellbird hepatoviruses were more closely related to viruses found in other species than to each (although with <90% bootstrap support). These results suggest that cross-species virus transmission has occurred in the recent evolutionary history of these avian hosts. This was supported by the reconciliation analysis that found cross-species transmission to be the most common co-phylogenetic process. Of note, the high diversity of avian viruses found in seemingly healthy passerines in our study and other studies worldwide (e.g. Truchado et al. 2020, Marzal et al. 2022, Shan et al. 2022) suggests that passerines may have a relatively high tolerance for viral infection, although this will need to be investigated further. Alternatively, the very high abundance values of the avian astroviruses and atadenovirus in the grey warbler suggests that some of the birds we sampled were in fact diseased, despite appearing healthy when sampled.

Passerines also make up a large proportion (47% of species) of the total avian community on Pukenui/Anchor Island, which may allow more viruses to be carried by these particular avian species, resulting in the relatively high diversity observed. Passerine birds were also observed foraging together in multispecies flocks on the Island (French et al. 2023), which could create more opportunities for cross-species transmission, and more viral sharing as a result of a similar diet. Although these multispecies flocks also included the Kākāriki (a parrot), we found no viruses directly shared between Kākāriki and any of the passerine species living in this restricted geographic area. This provides further evidence for the existence of a phylogenetic barrier to successful virus transmission, such that these hosts are too distantly related for viruses to easily jump between them (French et al. 2023). Thus, the behaviour that results in regular close contact, such as multispecies flocking, roosting (Ruiz-Aravena et al. 2022), or predator–prey interactions (Leendertz et al. 2008) appears to be a key driver of cross-species transmission, but only when the host species are sufficiently closely related to enable productive infections. Correspondingly, closely related species can inhabit the same physical environment but without regular close contact due to their distinct ecological niches (i.e. niche segregation; Young et al. 2010, Torretta et al. 2021). This would limit cross-species virus transmission and is likely the case with the parrot species on the Island. As a case in point, the Kākāpō is a ground-dwelling, herbivorous, nocturnal parrot which is unlikely to have frequent contact with the closely related Kākāriki or Kākā—both flighted, omnivorous, diurnal parrots. Niche segregation has also been suggested to drive the lack of cross-species viral transmission in New Zealand skink and gecko species (Waller et al. 2024).

Surprisingly, we only found one virus (a parvovirus) associated with a single parrot species on Pukenui/Anchor Island, and no host-infecting viruses in the other two parrot species. Several host-infecting viruses have been found in apparently healthy wild parrots in Australia (Sarker 2021) and China (Shan et al. 2022). Apart from a study of nesting material in yellow-crowned parakeets (Sikorski et al. 2013), this is the first metagenomic study of viruses in parrot populations in New Zealand. Thus, further work is needed to determine whether New Zealand parrot species truly have a low diversity of viruses. However, the gut microbiome of the Kākāpō (one of the species sampled in our study which had no host-infecting viruses) also has a very low microbial diversity (Waite et al. 2012, Perry et al. 2017). This may indicate that both the microbiome and virome lack diversity in Kākāpo, perhaps because of the severe historic population bottlenecks in this rare species (Dussex et al. 2021). It is also of note that as we only took cloacal samples (sampling tissues would be unethical), it is also possible that viruses are infecting these parrot species but not shedding via the digestive tract.

A number of viruses were found in the seabird species on the Island. The Tawaki/Fiordland crested penguin had a high diversity with host-infecting viruses from three different families, a viral diversity broadly similar to that in other penguin species (Wille et al. 2020). However, the viruses we identified were not closely related to other penguin viruses, but rather were related to viruses found in more distantly related host species (primarily waterbirds). This could either indicate past cross-species transmission from other birds into Tawaki, or reflect under-sampling across penguin species in New Zealand and worldwide.

The discovery of novel viruses expands our knowledge of avian viruses. For example, we identified two pegiviruses in the fernbird library. Until recently, pegiviruses had only been found in mammals, with the first non-mammalian pegivirus discovered in 2020 (Wu et al. 2020) in geese (*Anser cygnoides*). That the fernbird pegiviruses were related to geese pegiviruses suggests that there may be a larger clade of undiscovered avian pegiviruses. Unlike other pegiviruses, the goose pegiviruses are pathogenic, with infection resulting in reduced weight gain and 7% mortality (Wu et al. 2020). Although none of the animals caught for this study had overt signs of disease, it is possible that the closely related fernbird pegiviruses are also pathogenic, and more work is clearly needed to determine this.

Of particular note was the identification of a flavivirus in the brown creeper library that falls into the group of no-known-vector flaviviruses within the genus *Orthoflavivirus*. To our knowledge, this is the first virus found in birds that falls into this clade. Although we were only able to obtain a small proportion of its genome sequence, the phylogenetic position of the browncreeper flavivirus raises important questions as to its origin, pathogenicity and whether it can replicate in vertebrates, particularly as the orthoflaviviruses include a variety of arthropod-transmitted viruses that can be transmitted to vertebrates, often resulting in disease (McLean et al. 2021). While we found multiple flaviviruses in invertebrates on Pukenui/Anchor Island (French et al. 2023) including in mites, pseudoscorpions, and flies, they were all highly divergent with none that were closely related to the virus found in the browncreeper. However, as the virus was only found at low abundance (1.36 RPM), whether it was infecting the browncreeper or some component of its diet remains uncertain.

This study has implications for conservation management. Pukenui/Anchor Island is one of the most significant predator-free islands in New Zealand including populations of endemic forest and marine bird species. Some species, such as the Kākāpō and mohua, were translocated to the Island for conservation purposes. The Island populations of these species are vulnerable to infectious disease (Gartrell et al. 2005, Winter et al. 2022), such that identifying and mitigating threat of disease emergence is vital to recovery efforts. Potential viral transmission routes identified in this study (e.g. via passerines) will help guide conservation management actions on the Island, such as the supplementary feeding of Kākāpō and the introduction of other threatened species like the orange-fronted Kākāriki. For instance, management actions could include such measures as limiting or adjusting supplementary feeding practices to reduce points of contact between passerines and Kākāpō where viral transmission might occur. This is particularly relevant given that supplementary feeding stations could attract both Kākāpō and other birds, potentially increasing interspecies transmission risks. Additionally, understanding these routes is important when considering the introduction of other endangered species to the Island, such as the orange-fronted Kākāriki, as these interactions could introduce or amplify new viral risks to the ecosystem. Identifying these transmission pathways enables targeted biosecurity measures, enhancing the overall health management strategy for the Island’s ecosystem. Furthermore, the viral community will in turn be influenced by conservation management of the hosts, particularly when new host species are added to communities via translocation (French et al. 2022b). On a broader scale, island sanctuaries are a critical component of New Zealand’s successful conservation management strategy, without which many threatened species would now be extinct. Understanding the viral risks in these communities is important in protecting the threatened species they contain, particularly in the face of a growing threat from disease induced by climate change.

In sum, our investigation of the avian community on a remote New Zealand Island unveiled a high diversity of viruses, with evidence of cross-species transmission but only among passerine species. The prevalence of multispecies flocks and shared ecology among passerines likely contributes to this diversity and the occurrence of cross-species transmission. Expanding to include sampling over multiple time points would reveal whether seasonality impacts viral diversity among these taxa. Overall, this study emphasizes the need for continued exploration in diverse ecosystems to unravel the complexities of viral diversity and transmission dynamics, and is important in the conservation of threatened bird species that depend on island habitats.

## Supplementary Material

veae113_Supp

## Data Availability

The non-host sequence data generated in this study has been deposited in the Sequence Read Archive (SRA) under the accession number SAMN30927701-49. Virus consensus sequences have been deposited NCBI/GenBank with assigned accession numbers OQ986605–OQ986719.
