# Supplementary material for: Diversity and cross-species transmission of viruses in a remote island ecosystem: implications for wildlife conservation
Source: Virus Evol. 2024 Dec 14;11(1):veae113. doi: 10.1093/ve/veae113 (PMC11711479; doi:10.1093/ve/veae113)
Supplement: veae113_Supp [file veae113_supp.zip › suppl_data/French.Supplementary Figure 2.pdf]

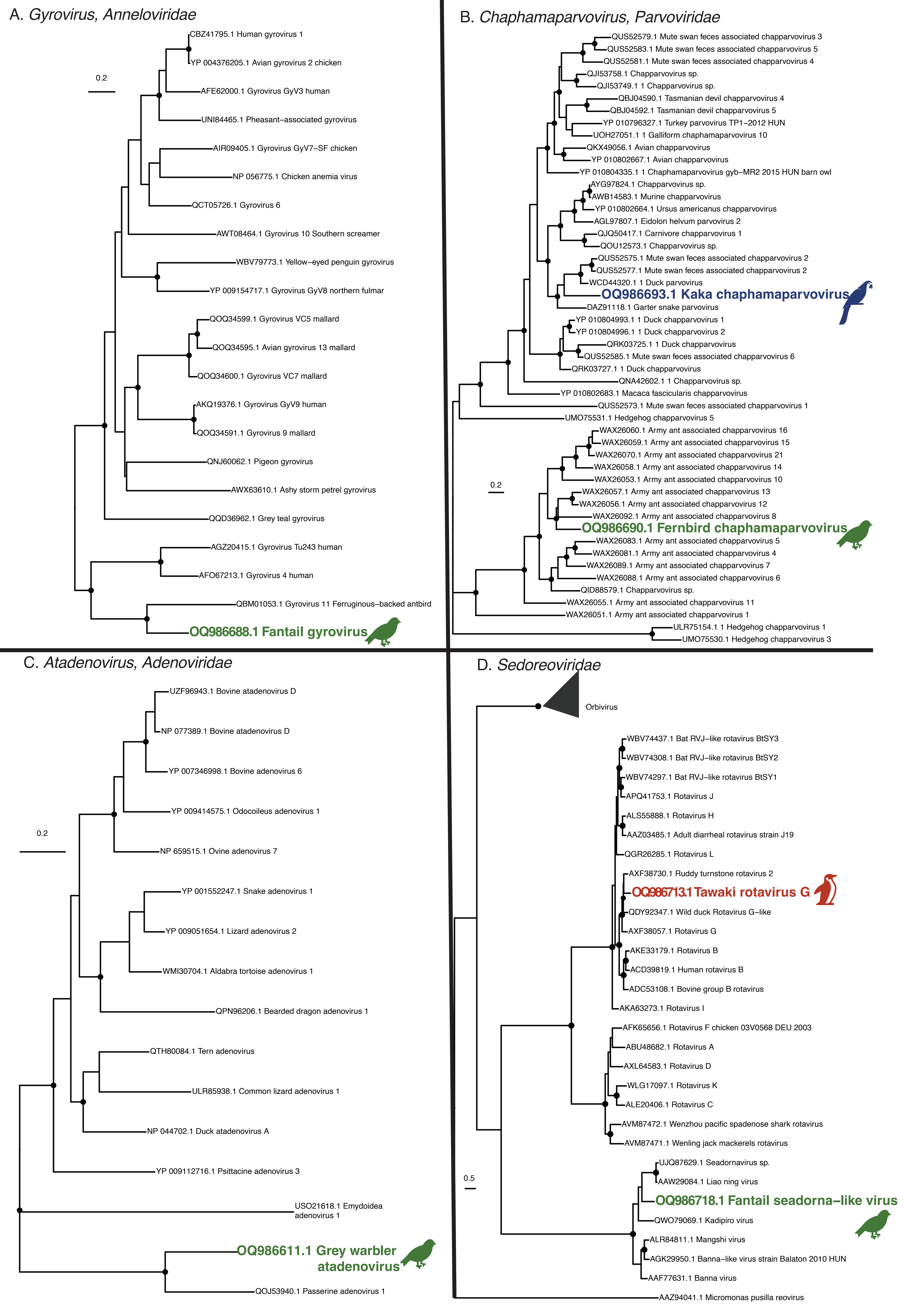

A. Gyrovirus, Anneloviridae
0.2
CBZ41795.1 Human gyrovirus 1
YP 004376205.1 Avian gyrovirus 2 chicken
AFE62000.1 Gyrovirus GyV3 human
UNI84465.1 Pheasant–associated gyrovirus
AIR09405.1 Gyrovirus GyV7–SF chicken
NP 056775.1 Chicken anemia virus
QCT05726.1 Gyrovirus 6
AWT08464.1 Gyrovirus 10 Southern screamer
WBV79773.1 Yellow–eyed penguin gyrovirus
YP 009154717.1 Gyrovirus GyV8 northern fulmar
QOQ34599.1 Gyrovirus VC5 mallard
QOQ34595.1 Avian gyrovirus 13 mallard
QOQ34600.1 Gyrovirus VC7 mallard
AKQ19376.1 Gyrovirus GyV9 human
QOQ34591.1 Gyrovirus 9 mallard
QNJ60062.1 Pigeon gyrovirus
AWX63610.1 Ashy storm petrel gyrovirus
QQD36962.1 Grey teal gyrovirus
AGZ20415.1 Gyrovirus Tu243 human
AFO67213.1 Gyrovirus 4 human
QBM01053.1 Gyrovirus 11 Ferruginous–backed antbird
OQ986688.1 Fantail gyrovirus
B. Chaphamaparvovirus, Parvoviridae
QUS52579.1 Mute swan feces associated chapparvovirus 3
QUS52583.1 Mute swan feces associated chapparvovirus 5
QUS52581.1 Mute swan feces associated chapparvovirus 4
QJI53758.1 Chapparvovirus sp.
QJI53749.1 1 Chapparvovirus sp.
QBJ04590.1 Tasmanian devil chapparvovirus 4
QBJ04592.1 Tasmanian devil chapparvovirus 5
YP 010796327.1 Turkey parvovirus TP1–2012 HUN
UOH27051.1 1 Galliform chaphamaparvovirus 10
QKX49056.1 Avian chapparvovirus
YP 010802667.1 Avian chapparvovirus
YP 010804335.1 1 Chaphamaparvovirus gyb–MR2 2015 HUN barn owl
AYG97824.1 Chapparvovirus sp.
AWB14583.1 Murine chapparvovirus
YP 010802664.1 Ursus americanus chapparvovirus
AGL97807.1 Eidolon helvum parvovirus 2
QJQ50417.1 Carnivore chapparvovirus 1
QOU12573.1 Chapparvovirus sp.
QUS52575.1 Mute swan feces associated chapparvovirus 2
QUS52577.1 Mute swan feces associated chapparvovirus 2
WCD44320.1 1 Duck parvovirus
OQ986693.1 Kaka chaphamaparvovirus
DAZ91118.1 Garter snake parvovirus
YP 010804993.1 1 Duck chapparvovirus 1
YP 010804996.1 1 Duck chapparvovirus 2
QRK03725.1 1 Duck chapparvovirus
QUS52585.1 Mute swan feces associated chapparvovirus 6
QRK03727.1 1 Duck chapparvovirus
QNA42602.1 1 Chapparvovirus sp.
YP 010802683.1 Macaca fascicularis chapparvovirus
QUS52573.1 Mute swan feces associated chapparvovirus 1
UMO75531.1 Hedgehog chapparvovirus 5
WAX26060.1 Army ant associated chapparvovirus 16
WAX26059.1 Army ant associated chapparvovirus 15
WAX26070.1 Army ant associated chapparvovirus 21
WAX26058.1 Army ant associated chapparvovirus 14
WAX26053.1 Army ant associated chapparvovirus 10
0.2
WAX26057.1 Army ant associated chapparvovirus 13
WAX26056.1 Army ant associated chapparvovirus 12
WAX26092.1 Army ant associated chapparvovirus 8
OQ986690.1 Fernbird chaphamaparvovirus
WAX26083.1 Army ant associated chapparvovirus 5
WAX26081.1 Army ant associated chapparvovirus 4
WAX26089.1 Army ant associated chapparvovirus 7
WAX26088.1 Army ant associated chapparvovirus 6
QID88579.1 Chapparvovirus sp.
WAX26055.1 Army ant associated chapparvovirus 11
WAX26051.1 Army ant associated chapparvovirus 1
ULR75154.1 1 Hedgehog chapparvovirus 1
UMO75530.1 Hedgehog chapparvovirus 3
C. Atadenovirus, Adenoviridae
UZF96943.1 Bovine atadenovirus D
NP 077389.1 Bovine atadenovirus D
YP 007346998.1 Bovine adenovirus 6
YP 009414575.1 Odocoileus adenovirus 1
0.2
NP 659515.1 Ovine adenovirus 7
YP 001552247.1 Snake adenovirus 1
YP 009051654.1 Lizard adenovirus 2
WMI30704.1 Aldabra tortoise adenovirus 1
QPN96206.1 Bearded dragon adenovirus 1
QTH80084.1 Tern adenovirus
ULR85938.1 Common lizard adenovirus 1
NP 044702.1 Duck atadenovirus A
YP 009112716.1 Psittacine adenovirus 3
USO21618.1 Emydoidea adenovirus 1
OQ986611.1 Grey warbler atadenovirus
QOJ53940.1 Passerine adenovirus 1
D. Sedoreoviridae
Orbivirus
WBV74437.1 Bat RVJ–like rotavirus BtSY3
WBV74308.1 Bat RVJ–like rotavirus BtSY2
WBV74297.1 Bat RVJ–like rotavirus BtSY1
APQ41753.1 Rotavirus J
ALS55888.1 Rotavirus H
AAZ03485.1 Adult diarrheal rotavirus strain J19
QGR26285.1 Rotavirus L
AXF38730.1 Ruddy turnstone rotavirus 2
OQ986713.1 Tawaki rotavirus G
QDY92347.1 Wild duck Rotavirus G–like
AXF38057.1 Rotavirus G
AKE33179.1 Rotavirus B
ACD39819.1 Human rotavirus B
ADC53108.1 Bovine group B rotavirus
AKA63273.1 Rotavirus I
AFK65656.1 Rotavirus F chicken 03V0568 DEU 2003
ABU48682.1 Rotavirus A
AXL64583.1 Rotavirus D
WLG17097.1 Rotavirus K
ALE20406.1 Rotavirus C
AVM87472.1 Wenzhou pacific spadenose shark rotavirus
AVM87471.1 Wenling jack mackerels rotavirus
UJQ87629.1 Seadornavirus sp.
0.5
AAW29084.1 Liao ning virus
OQ986718.1 Fantail seadorna–like virus
QWO79069.1 Kadipiro virus
ALR84811.1 Mangshi virus
AGK29950.1 Banna–like virus strain Balaton 2010 HUN
AAF77631.1 Banna virus
AAZ94041.1 Micromonas pusilla reovirus
