# Supplementary figures and images for: Diversity and cross-species transmission of viruses in a remote island ecosystem: implications for wildlife conservation
Source: Virus Evol. 2024 Dec 14;11(1):veae113. doi: 10.1093/ve/veae113 (PMC11711479; doi:10.1093/ve/veae113)

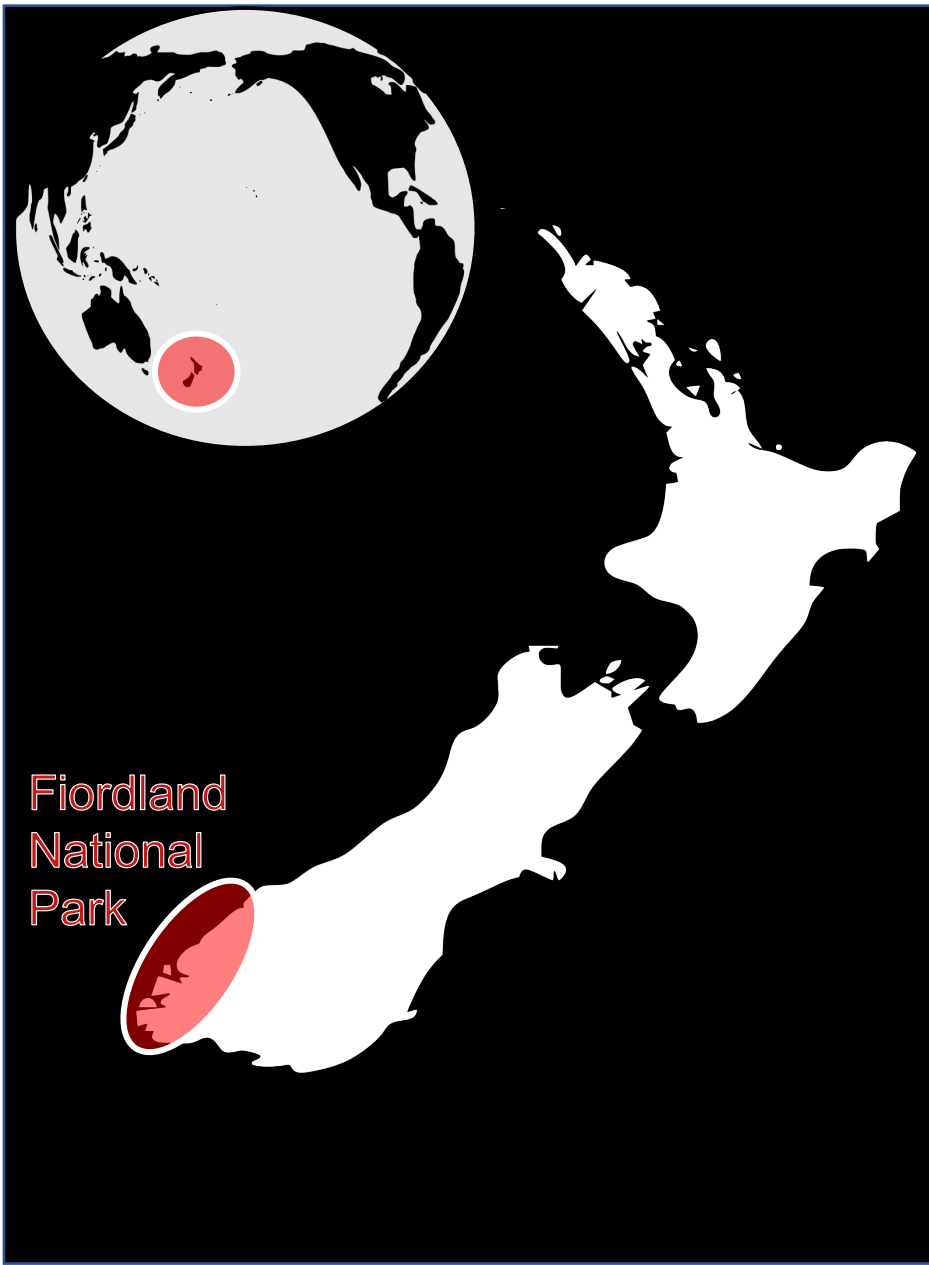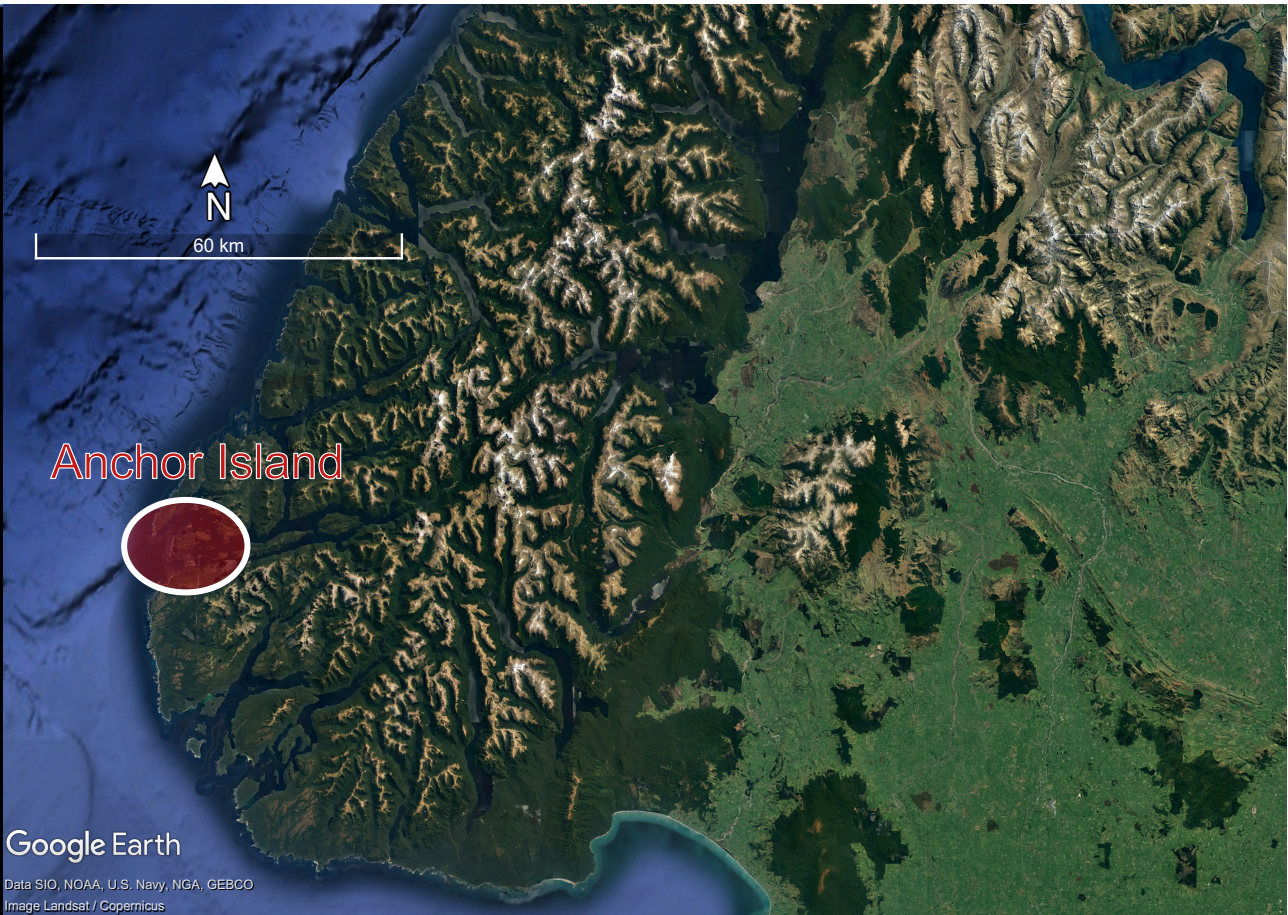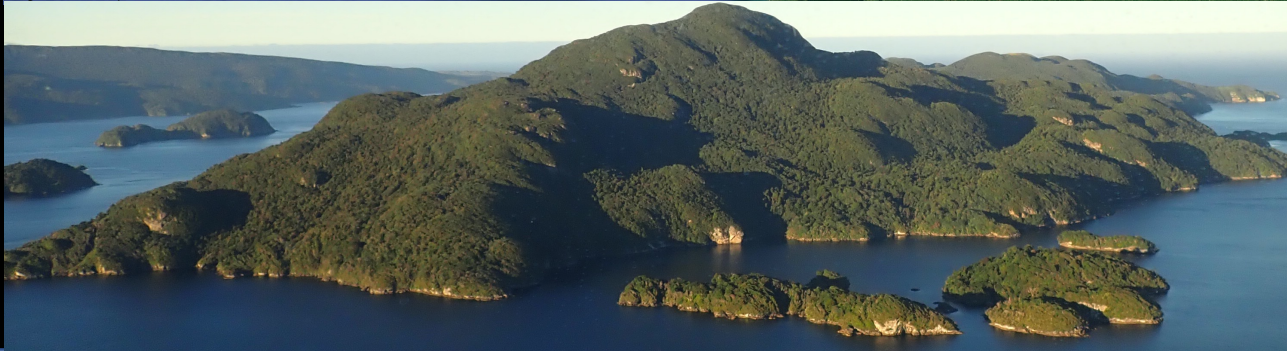

Supplement: veae113_Supp [file veae113_supp.zip › suppl_data/French.Supplementary Figure 1.pdf]
